# Dual Targeting of PTP1B and Aldose Reductase with Marine Drug Phosphoeleganin: A Promising Strategy for Treatment of Type 2 Diabetes

**DOI:** 10.3390/md19100535

**Published:** 2021-09-24

**Authors:** Massimo Genovese, Concetta Imperatore, Marcello Casertano, Anna Aiello, Francesco Balestri, Lucia Piazza, Marialuisa Menna, Antonella Del Corso, Paolo Paoli

**Affiliations:** 1 Department of Experimental and Clinical Biomedical Sciences, University of Florence, Viale Morgagni 50, 50134 Florence, Italy; m.genovese13@gmail.com; 2 Department of Pharmacy, University of Naples Federico II, Via D. Montesano 49, 80131 Napoli, Italy; cimperat@unina.it (C.I.); marcello.casertano@unina.it (M.C.); aiello@unina.it (A.A.); 3 Biochemistry Unit, Department of Biology, University of Pisa, Via S. Zeno, 51, 56123 Pisa, Italy; francesco.balestri@unipi.it (F.B.); l.piazza@studenti.unipi.it (L.P.)

**Keywords:** marine natural products, metabolic diseases, type 2 diabetes mellitus, protein tyrosine phosphatase 1B, aldose reductase, multitarget drugs

## Abstract

An in-depth study on the inhibitory mechanism on protein tyrosine phosphatase 1B (PTP1B) and aldose reductase (AR) enzymes, including analysis of the insulin signalling pathway, of phosphoeleganin, a marine-derived phosphorylated polyketide, was achieved. Phosphoeleganin was demonstrated to inhibit both enzymes, acting respectively as a pure non-competitive inhibitor of PTP1B and a mixed-type inhibitor of AR. In addition, in silico docking analyses to evaluate the interaction mode of phosphoeleganin with both enzymes were performed. Interestingly, this study showed that phosphoeleganin is the first example of a dual inhibitor polyketide extracted from a marine invertebrate, and it could be used as a versatile scaffold structure for the synthesis of new designed multiple ligands.

## 1. Introduction

Designing multitarget drugs (agents that can simultaneously interact with multiple biological entities) for the treatment of multifactorial pathologies is a promising approach and a new challenge in medicinal chemistry [1]. Single-target drugs, indeed, have been clearly demonstrated to be inadequate to achieve a therapeutic effect in the case of some complex multifactorial diseases [2,3]. On the other hand, it is now clear that many drugs may derive their therapeutic effect (as well as their undesirable off-target activities) from the interactions with multiple targets and, thus, it looks as if many clinically useful drugs are multitarget agents. Therefore, we are moving beyond the traditional one-target-one-drug model in drug discovery to a more effective strategy, in which compounds with a well-defined multitarget profile are addressed and rationally designed. These are the so-called designed multiple ligands (DMLs), well-distinguished from nonselective agents with activity against targets not involved in the selected disease, being designed and developed to achieve highly specific modulation of two or more selected biological targets [4,5,6,7].

A multitarget approach could be particularly beneficial for treatment of type 2 diabetes mellitus (T2DM), which is increasing at an alarming rate, owing to incorrect lifestyle and dietary habits, in addition to population ageing [8,9,10]. T2DM is a multifactorial chronic disease characterised by hyperglycaemia, originating from reduced sensitivity of target tissues to insulin (insulin resistance), and other metabolic dysfunctions. It is hyperglycaemia, indeed, that triggers abnormal cellular signalling and induces cellular dysfunctions, which are, in turn, responsible for physical damages, failure of different organs, and serious chronic complications, including nephropathy and cardiovascular diseases (CVD). Moreover, a relationship between T2DM and obesity has been recognised, and the major basis for this link is the ability of obesity to engender insulin resistance [11]. Nowadays, effective therapeutics are currently available to control hyperglycaemia and prevent chronic complications in diabetic patients; however, in most cases, combinations of various drugs are required, leading to drug–drug interactions and inadequate patient adherence. The identification of common pathways as drug targets could surely be useful to determine molecules with a multitarget profile able to interact with them to contrast the disorder.

Recently, the challenge of developing small-molecule inhibitors of protein tyrosine phosphatase 1B (PTP1B) has resulted in intense research; it can be considered a novel target for T2DM and its inhibition as a point of intervention could provide a new therapeutic option to patients with at-risk obesity or T2DM. PTP1B is crucially implicated in the development of insulin resistance; it is a negative regulator of insulin action, by dephosphorylating specific residues of phosphotyrosine (pTyr) of the activated insulin receptor (IR) and, thus, interrupting the signalling pathways mediated by the hormone. Among the insulin target organs, adipose tissue reacts to endocrine and metabolic signals by either increasing or decreasing a variety of adipokines and cytokines that can act at both the local and systemic level [12]. PTP1B also downregulates the signal of leptin, an adipokine that controls food intake and increases energy expenditure and, thus, has a central importance in the global obesity and cardiovascular disease problem [13]. PTP1B overexpression is strictly related to insulin resistance, whereas PTP1B inhibition or genetic ablation improve glucose homeostasis, cellular sensitivity to both insulin and leptin, and resistance to diet-induced obesity, without inducing hypoglycaemia or toxic effects [14]. The efforts made in recent decades have allowed the identification of new inhibitors, which prove to be well tolerated and effective, both as insulin-sensitising agents and as antidiabetic drugs in animal models of T2DM [15]. Together, this evidence confirms that development of potent and safe PTP1B inhibitors could represent one of the most interesting challenges for the discovery of new therapeutic interventions in T2DM and its complications in the coming years.

Aldose reductase (AR) has become another potential drug target for developing new therapeutics for treatment of T2DM and its complications. It is a cytosolic enzyme involved in the polyol pathway, in which glucose is converted to sorbitol that is, in turn, transformed in fructose through the NAD^+^-dependent action of sorbitol dehydrogenase. In hyperglycaemic conditions, due to saturation of the glycolytic enzyme hexokinase, an increased flux of glucose is channelled through the polyol pathway, leading to several metabolic alterations. In particular, the accumulation of the osmolyte sorbitol increases cellular osmolarity, inducing a deleterious cell swelling. At the same time, the advanced glycation end product formation, linked to increased fructose levels, promotes ROS generation and NF-κB expression, leading to oxidative stress. In addition, the NADPH depletion linked to AR activity impairs the glutathione-reductase-dependent recovery of reduced glutathione. On the other hand, the increase in NADH/NAD^+^ ratio might lead to NADH oxidase upregulation, thus further promoting oxidative stress. Under these conditions, lipid peroxidation occurs, with the consequent formation of cytotoxic aldehydes, including 4-hydroxy-*trans*-2-nonenal and its derivative conjugated with glutathione (GS-HNE). AR reduces GS-HNE in the corresponding alcohol (3-glutathionyl-1,4-dihydroxynonane), which promotes the expression of NF-κB. In conclusion, the enhanced activity of AR promotes not only the onset of oxidative stress linked to insulin resistance, but also the long-term complications linked to T2DM [16,17]. Based on the above, the potential of facing the complex and multifactorial T2DM disease by combining two selective inhibitors into one molecule with dual PTP1B and AR modulator activity is evident. A dual inhibitor of the PTP1B and AR enzymes could be useful to treat both the condition of insulin resistance and chronic complications associated with T2DM [18,19,20].

T2DM is spreading rapidly, not only in industrialised countries, but also in developing ones, where the economic resources available to tackle this pandemic are very limited [21]. Most people living in these countries rely on remedies of traditional medicine to counteract metabolic alterations induced by diabetes [22]. Such extracts obtained using different parts of plants, algae, and terrestrial or aquatic organisms contain a plethora of natural molecules able to act as multiple target ligands [23]. Therefore, natural products (NPs), used either unaltered or developed through chemical manipulation and synthesis, represent one of the most important sources of scaffold molecules showing innate multitarget activity that could be used for the development of new DMLs [24,25]. The marine environment, in particular, offers a chemical arsenal of unique structures with different structural motifs compared to NPs of terrestrial origin because of the different conditions of salinity, pressure, and temperature that influence biosynthetic pathways [26,27,28]. Several NPs of marine origin have been reported as inhibitors of both PTP1B and AR enzymes; they include terpenes, polyketides, steroids, phenols, and alkaloids, mainly isolated from marine invertebrates [29,30,31,32].

**Figure 1:**
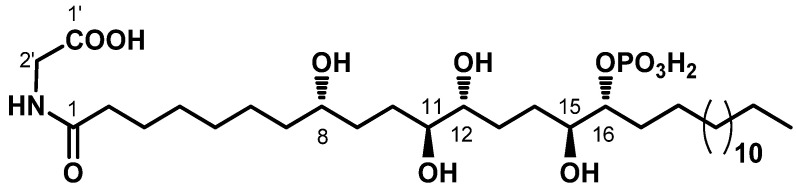
Structure of the marine-derived polyketide phosphoeleganin (PE).

Recently, we have isolated from the Mediterranean ascidian *Sidnyum elegans* a novel phosphorylated compound and potent PTP1B inhibitor, phosphoeleganin (PE, Figure 1) [33,34]. 

In order to explore the potential of PE to be developed as a multitarget drug to treat insulin resistance and chronic complications associated with T2DM, we have carried out an in-depth study on its inhibitory mechanism, including analysis of the insulin signalling pathway; in parallel, we have evaluated the AR inhibitory activity of PE. The results reported here show that PE inhibits both enzymes, acting, respectively, as a mixed-type inhibitor of AR and a pure non-competitive inhibitor of PTP1B. In addition, in silico docking analyses were performed to describe how PE interacts with both enzymes. Finally, tests carried out using HepG2 cells confirmed that PE possesses an insulin-sensitising activity. 

## 2. Results

### 2.1. Chemistry

PE was obtained in pure form (Figures S1 and S2) by extraction of a sample of the Mediterranean ascidian *S. elegans* from the existing collection of natural sources available at the Department of Pharmacy of University of Naples Federico II. PE was isolated and purified according to the previously described procedure [33] and unequivocally identified by comparison of its HRMS and NMR data with those reported in the literature [33].

### 2.2. Preliminary Screening on PTP1B and AR In Vitro

To assess the effect of PE on PTP1B and AR catalytic activities, a preliminary evaluation of IC_50_ was performed. The obtained results confirm the ability of PE to inhibit PTP1B and highlight that PE can also act as an AR inhibitor, despite a minor efficiency (IC_50_ = 28.7 ± 1.1 μM). The IC_50_ value determined for PTP1B (1.3 ± 0.04 μM) is almost eight times lower than that previously determined [33]. We think that such differences may be attributable to the different experimental conditions used to perform the activity assays, such as a different pH value and the use of a GST–PTP1 fusion protein instead of purified PTP1B alone (1-302 aa).

### 2.3. Definition of PTP1B and AR Inhibition Mechanism by PE 

**Figure 2:**
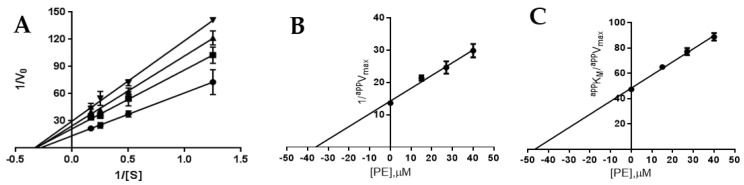
Kinetic characterisation of PE as an AR inhibitor. Panel (**A**) AR activity (10 mU) at the indicated L-idose concentrations in the presence of PE at concentrations: 0 (●), 15 (■), 27 (▲), and 40 µM (▼). Data are reported as a Lineweaver–Burk plot. Bars (when not visible, are within the symbols’ size) represent the standard deviations of the means from at least three independent measurements. Panels (**B**) and (**C**) Secondary plots of the ordinate intercepts (1/*^app^V_max_*) and of the slopes (*^app^K_M_*/*^app^V_max_*), respectively, of the primary plot as a function of PE concentration. Bars (when not visible, are within the symbols’ size) represent the standard error of the ordinate intercepts and of the slopes obtained from the data of Panel A.

The mechanism of the enzyme inhibition by PE was investigated and it was shown that it behaves as a reversible inhibitor of both AR and PTP1B. As far as AR is concerned, indeed, we found that 100% of enzyme activity was recovered after the inhibitor had been removed by extensive dialysis (see Methods for details). Analogously, dilution assay test showed that PTP1B recovered its activity almost completely after incubation with a saturating concentration of PE, thereby confirming that PE behaves as a reversible inhibitor of PTP1B (Figures S3 and S4). Kinetic characterisation of PE as an AR and PTP1B inhibitor was carried out. Kinetic analysis revealed that PE acts as a mixed inhibitor of AR (Figure 2A), being able to affect to a different extent both *V_max_* and *K_M_.* The analysis of the dependence of *^app^V_max_* and *^app^K_M_*/*^app^V_max_* on the inhibitor concentration allowed the measurement of *K’_i_* (36 ± 1 μM, Figure 2B) and *K_i_* (47 ± 1 μM, Figure 2C).

**Figure 3:**
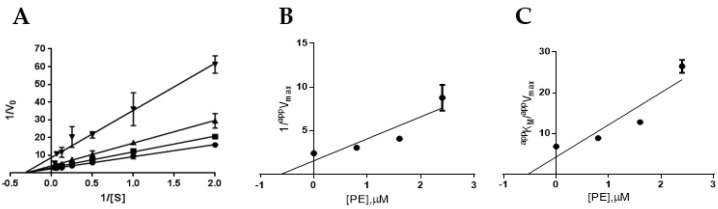
Kinetic characterisation of PE as a PTP1B inhibitor. (**A**) PTP1B activity (8 mU) at the indicated *p*-NPP concentrations in the presence of PE at concentrations: 0 (●), 0.8 (■), 1.6 (▲), and 2.4 µM (▼). Data are reported as a Lineweaver–Burk plot. Bars (when not visible, are within the symbols’ size) represent the standard deviations of the means from at least three independent measurements. (**B**) and (**C**) Secondary plots of the ordinate intercepts (1*/^app^V_max_*) and of the slopes (*^app^K_M_*/*^app^V_max_*), respectively, of the primary plot as a function of PE concentration. Bars (when not visible, are within the symbols’ size) represent the standard error of the ordinate intercepts and of the slopes obtained from the data of Panel A.

These values indicated a slight, but significant (*p* < 0.001), preference of PE toward the ES complex with respect to the free enzyme. On the other hand, PE acts as a pure non-competitive inhibitor of PTP1B; the analysis of the Lineweaver–Burk plot, indeed, indicated that only *V_max_* is affected by the presence of the inhibitor (Figure 3). In fact, the analysis of the dependence of *^app^V_max_* and *^app^K_M_*/*^app^V_max_* on the inhibitor concentration allowed the measurement of an identical value of 0.7 ± 0.1 µM for *K_i_* and *K’_i_*_._

### 2.4. Dissecting the Interaction of PE with PTP1B and AR by In Silico Docking Analyses

**Figure 4:**
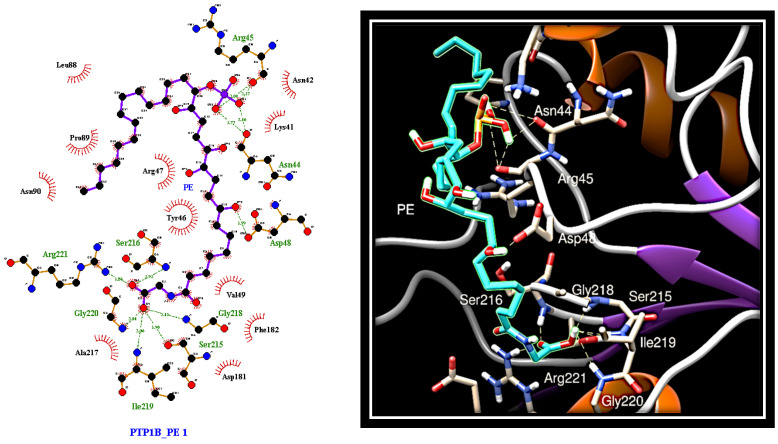
Docking analysis revealing the most favourable interaction of PE with PTP1B. Left: protein-ligand interactions of PE (in purple) bound to PTP1B. Red circles: Lipophilic contacts; green dotted lines: hydrogen bonds. Right: 3D image of the selected pose of PE (in cyan) bound to PTP1B. Grey dotted lines: hydrogen bounds.

To evaluate the interaction mode of PE with PTP1B, in silico docking analysis was carried out using AutoDock software. The compound was docked to the whole protein surface of PTP1B. We found that PE was able to fit to different sites that can be categorised depending on the docking energy. The most favourable position places PE between the active site and the region that defines the “C” site [35], which differs from the secondary aryl phosphate site (identified by Arg24 and Arg254) previously described as the docking site for aryl difluorophosphonate groups (Figure 4) [36]. 

Interestingly, the phosphate group of PE creates hydrogen bonds with the carbonyl groups of Asn44 and Arg45 that lie beyond the residues forming the "C" site. This suggests an alternative bonding mode of PE on the surface of PTP1B compared to fluorine-bisphosphonates. Further hydrogen bonds involve the Asp48 residue belonging to the “C” site and a hydroxyl group present on the aliphatic chain (C12) of PE. Finally, the carbon chain of PE extends to the active site where the carboxyl group of PE forms several hydrogen bonds with the side chain of Arg221 and the nitrogen atoms of the main chains of Ser215, Ser216, Gly218, Ile219, and Gly220.

**Figure 5:**
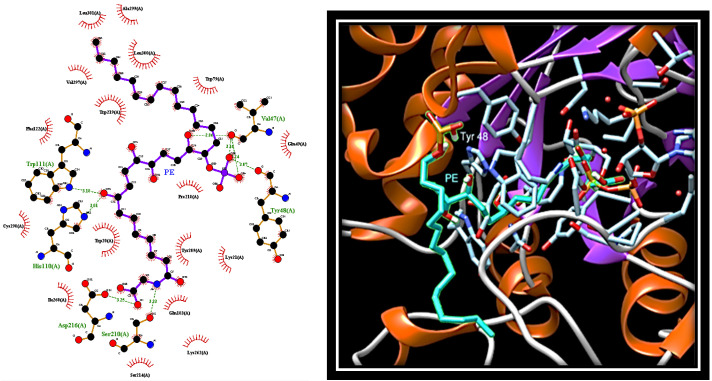
Docking analysis revealing the most favourable interaction of PE with AR. Left: protein-ligand interactions of PE (in purple) bound to AR. Red circles: Lipophilic contacts; green dotted lines: hydrogen bonds. Right: 3D image of the selected pose of PE (in cyan) bound to AR. Grey dotted lines: hydrogen bounds.

The docking analysis carried out in the absence of substrate localises PE in the active site of AR (Figure 5). 

Here, the OH group at C8 forms hydrogen bonds with nitrogen atoms of His110 and Trp111, whereas the OH at C15, as well as the phosphate group, form hydrogen bonds with carbonyl groups of the main chain of Val47 and Tyr48. In addition, the aliphatic portion of PE (C17-30) is firmly anchored to the selectivity pocket, where it makes several hydrophobic interactions with Trp79, Trp219, Val297, Ala299, Leu300, and Leu301. Finally, the portion from the carboxyl group at C7 interacts with Trp20 and Tyr209, and extends up to a more hydrophilic region where the carboxyl group forms hydrogen bonds with side chains of Asp216 and Ser210.

**Figure 6:**
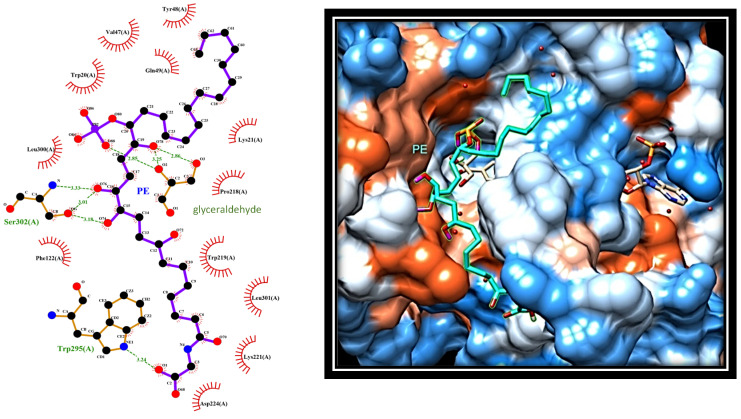
Docking analysis revealing the most favourable interaction of PE with AR in the presence of substrate (glyceraldehyde) and NADPH. Left: protein-ligand interactions of PE (in purple) bound to AR. Red circles: Lipophilic contacts; green dotted lines: hydrogen bonds. Right: 3D image of the selected pose of PE (in cyan) bound to AR active site.

When PE was docked in the AR–substrate complex, the interaction with the enzyme appeared different, since it involved amino acids located in the external portion of the active site (Figure 6). 

In this arrangement, the terminal carboxyl portion of PE (C7–C1’) extends out of the active site, making a hydrophobic interaction with Trp219, Leu301, and Lys221, while the carboxyl group of PE form a hydrogen bond with the nitrogen atom of Trp295. On the other side, the hydroxyl groups at both C15 and C16 face the mouth of the active site, where they form hydrogen bonds with the oxygen atom of Ser302. In addition, phosphate moiety moves into the active site, where it forms hydrogen bonds with glyceraldehyde. Finally, the C17–C30 portion of PE remains excluded from the active site, interacting with amino acids surrounding the entrance to the active site, including the aliphatic chain of Lys21, Tyr48, and Gln49. These data concur with kinetic analysis in confirming the ability of PE to efficiently interact with AR.

### 2.5. Analysis of the Insulin Signalling Pathway in Liver Cells Treated with PE

**Figure 7:**
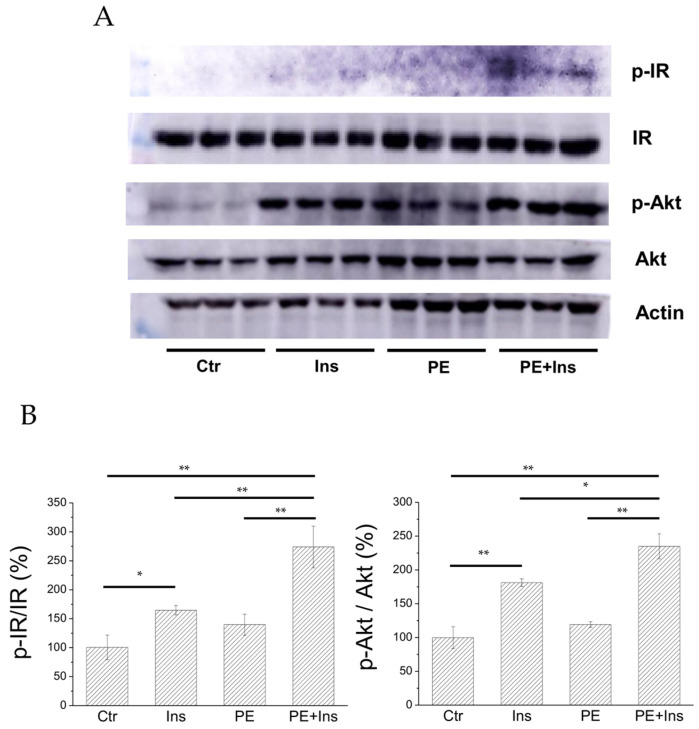
Effect of PE on the insulin signalling pathway. Panel **A**: Western blot image; Panel **B**: quantitation of Western blot carried out using Kodak MI software. Data shown were normalized with respect to the control sample. All tests were carried out in triplicate. Statistical analysis was repeated using ANOVA, followed by Tukey HSD. Data were checked for appropriate normality and homoscedasticity using the Shapiro–Wilk normality test and the Levene’s test, respectively. Ctr: control experiment; Ins: cells treated with 10 nM insulin; PE: cells treated with compound PE (25 μM); * *p* < 0.05; ** *p* < 0.01.

To evaluate whether PE is able to improve the insulin signalling pathway in vitro, we analysed the effect of the compound on HepG2 cell line. For this purpose, we treated HepG2 cells with PE (25 µM final concentration) for 30 minutes in the presence or absence of insulin (10 nM). After treatment, cells were lysed and cellular extracts were analysed to evaluate the phosphorylation status of insulin receptor (IR) and Akt, a kinase downstream IR (Figure 7). 

We found that, in liver cells treated with PE alone, the phosphorylation levels of both IR and Akt are similar to those detected in control cells, suggesting that PE did not behave as an insulin-mimetic agent. Nevertheless, a significant increase in IR and Akt phosphorylation levels was observed, combining insulin stimulation with PE treatment. Taken together, these results demonstrated that PE acts as an insulin-sensitising agent.

## 3. Discussion

Diabetic patients may rely on different types of oral hypoglycaemic drugs to keep blood glucose below physiological levels; however, most of these drugs cause patients to develop various and serious diabetes-related diseases in the medium- to long-term that contribute to deterioration of the quality and duration of their life [37]. This is probably because actual antihyperglycemic drugs are designed to compensate for a single metabolic defect and, therefore, are not able to reproduce the physiological effects of insulin. Although the synthesis of DMLs is a very exciting challenge, the identification of the appropriate scaffolds represents the real greatest challenge in this approach because of the difficulty to obtain multifunctional molecules showing a balanced activity toward the selected targets [5]. Therefore, many researchers are concretely exploring the possibility of exploiting natural molecules, even of marine origin, to discover new DMLs to be used for the treatment of T2DM [29,30,31,32,33]. 

Here, we demonstrated that PE, a phosphorylated polyketide isolated from the Mediterranean ascidian *S. elegans*, behaves as a multitarget ligand, targeting both PTP1B and AR. The ability of PE to inhibit PTP1B was previously described [33]; however, its mechanism of action and its in vitro activity had not yet been studied. Being endowed with a phosphate group, we expected that PE could bind to the active site of PTP1B, acting as a classical competitive inhibitor. However, unexpectedly, our results showed that PE acts as a pure non-competitive inhibitor of PTP1B, showing a Ki value of 0.7 µM. Interestingly, in silico docking analyses suggested that the nonfunctionalised aliphatic portion of PE structure interacts with various hydrophobic residues present on the surface of the enzyme, whereas the phosphate group binds firmly to some amino acids close to the "C" site, a region previously described to act as a binding site for negatively charged difluorophenylmethylene phosphonates [35]. The other functionalised part of the molecule forms hydrogen or hydrophobic bonds with numerous other amino acids, extending to the area of the active site, where the carboxyl group of the PE forms further bonds with the residues of the P-loop. Therefore, for the first time, we investigated the way in which PE interacts with PTP1B, also clarifying the role of the phosphate group in stabilising the enzyme–inhibitor complex. Concerning AR, it is worth noting the effort in the last decades in synthetising AR inhibitors (ARI), with the aim to treat secondary complications related to diabetes [38]. Despite the proved clinical efficacy of some of these, the main limitation to their use in clinic is the severe side effects they induce [20]. Even if the identification of new and safe ARI remains one of the priority objectives in the pharmaceutical industry, the interest for ARIs with a well-defined multitarget profile has grown in the last years, based on the belief that they could have greater efficacy and less toxic effects than traditional ARI. The ability of PE to inhibit AR had not been reported until now. We showed, for the first time, that PE acts as a mixed-type inhibitor of AR (IC_50_ of 28.7 µM), being able, in the absence of substrate, to move inside the active site of enzyme or, in the presence of substrate, to interact on the upper part of the AR active site, positioning the phosphate group at the entry of the active site. In this position, the phosphate can interact with substrate, thereby interfering with the catalytic process.

Tests performed on liver cells showed that, after short-term PE treatment, the phosphorylation levels of IR and Akt kinase increased in hormone-stimulated, but not in non-stimulated, cells. Akt kinase is an essential effector of the insulin signalling pathway and its enhanced phosphorylation is an expected/essential event to propagate the signalling wave trigger by insulin [39]. Similarly, a plethora of studies demonstrated that the enhancement of Akt phosphorylation is a common landmark observed in both muscle and liver cells treated with PTP1B inhibitors [40,41,42,43]. In analogy with this evidence, the data reported in this study show that treatment of liver cells with PE enhances insulin activity, suggesting that it acts as an insulin-sensitising agent. Moreover, our data indicate that PE, despite the presence of a charged phosphate group, can cross the plasma membrane and move within the cytoplasm of cells where it inhibits PTP1B, thus enhancing the insulin signalling pathway. This finding is quite surprising, considering that most low molecular weight inhibitors with a phosphate group show poor permeability across the cell membrane [44]. Although no information is currently available on the mechanisms promoting PE internalisation in liver cells, it can be hypothesised that its entry into cells may be mediated by fatty acid transporters present on the cell surface or, alternatively, by the process of endocytosis. Further tests will be needed in the future to confirm this hypothesis. 

## 4. Material and Methods

### 4.1. Materials

Solvents and commercial reagents: Sigma-Aldrich (Saint Louis, MO, USA). TLC: Silica Gel 60 F254, plates 5 × 20, 0.25 mm, Merck (Kenilworth, NJ, USA). Deuterated solvents: Sigma-Aldrich-Merck. High-resolution MS (negative mode) was performed on a Thermo LTQ Orbitrap XL mass spectrometer (Thermo-Fisher, San Josè, CA, USA). The MS spectrum of PE was recorded by infusion into the ESI source using MeOH as solvent. ^1^H (700 MHz) and ^13^C (175 MHz) NMR were carried out on a Bruker Avance Neo spectrometer (Bruker BioSpin Corporation, Billerica, MA, USA); chemical shifts were referenced to the residual solvent signal (CD_3_OD: *δ*_H_ = 3.31, *δ*_C_ = 49.0). A Jasco P-2000 polarimeter (Jasco Europe s.r.l., Cremella, Italy) at the sodium D line was used to measure optical rotations. High-performance liquid chromatography (HPLC) separation was achieved on a Knauer K-501 apparatus equipped with a Knauer K-2301 RI detector (LabService Analytica s.r.l., Anzola dell’Emilia, Italy).

L-idose and NADPH were from Carbosynth (Compton, England); *p*-nitrophenylphosphate (*p*-NPP) was from Chemcruz, Santa Cruz Biotechnology (Santa Cruz, CA, USA). All other chemicals were obtained from Merck Life Science s.r.l. (St. Louis, MO, USA), unless otherwise specified. Cell culture media and foetal bovine serum (FBS) were from Euroclone (Pero, Italy). The following antibodies were used: pIR β subunit, Y1162/1163 (sc-25103-R) and β-actin, clone C-4 (sc-47778) were from Santa Cruz Biotechnology (Santa Cruz, CA, USA); IR β subunit, clone CT-3 (MABS65) was from Merck-Millipore (Burlington, MA, USA). Akt (9272S) and p-Akt (9271S) antibodies were from Cell Signaling Technology (Danvers, MA, USA). Secondary antibodies were from Santa Cruz Biotechnology (Santa Cruz, CA, USA).

The antibodies used were purchased by Merck (anti-insulin receptor, MABS65), Santa Cruz Biotechnology (p-insulin Rβ, sc-81500; β-actin, sc-47778), and Cell Signaling (Akt, 9272S; p-Akt, 9271S). Primary antibodies were diluted 1:1000 in PBS solution containing 0.05% Tween 20 and 5% BSA; secondary antibodies were diluted 1: 2500 in the same solution. Primary antibodies were incubated with PVDF membrane overnight at 4 °C; secondary antibodies were incubated for 1 hour at room temperature. Detection was performed using Clarity Western ECL substrates (Bio-Rad Laboratories S.r.l., Segrate, Milan, Italy).

### 4.2. Collection, Extraction, and Isolation of PE from Specimens of S. elegans

Specimens of *S. elegans* were collected at Pozzuoli (Naples, Italy, April 2019). They were frozen immediately after collection and kept frozen until extraction. Fresh thawed animals (21.3 g of dry weight after extraction) were extracted according to a previously reported procedure [33]. The most polar organic layer (n-BuOH material) was chromatographed by MPLC over a C-18 column following a gradient elution H_2_O → MeOH → CHCl_3_. The fraction eluted with MeOH/H_2_O 7:3 (*v*/*v*) was further purified by HPLC on reversed phase (column: Synergi RP-MAX 4 μm, eluent: MeOH/H_2_O 8:2 + 0.1% of TFA) and afforded compound PE (t_R_ = 20.5 min, 15.8 mg) in pure form. 

Phosphoeleganin (PE): colourless oil; all data agree with those reported in the literature [33,34].

### 4.3. Expression and Purification of AR and PTP1B

The human recombinant AR was expressed in BL21(DE3) pLysS *E. coli* cells and purified to electrophoretic homogeneity, as previously described [45]. Purified AR (5.0 U/mg) was stored at −80 °C in 10 mM sodium phosphate buffer pH 7.0 containing 2 mM dithiothreitol and 30 % (*w/v*) glycerol; the enzyme was extensively dialysed against 10 mM sodium phosphate buffer pH 7.0 before use. 

The human recombinant PTP1B (1-302 aa) and LMW-PTP (full-length) were expressed as fusion proteins containing *N*-terminus *polyhistidine tags* in BL21(DE3) pLysS *E. coli* and purified by using affinity chromatography. After purification, enzymatic assays were carried out to identify the fraction containing the enzyme. Such fractions were collected and concentrated using centrifugal filters. Finally, concentrate solution was purified using an FPLC system equipped with a Superdex 200 pg 16/600 semi-preparative column equilibrate in 50 mM Tri-HCl buffer pH 8.0 containing 150 mM NaCl, 1 mM DTT. Fractions containing the enzyme were collected, concentrated, and batched in cryovials that were stored at −80 °C. The degree of purity of the enzymes was evaluated by SDS PAGE. 

### 4.4. Determination of Enzymatic Activities

AR activity was determined at 37 °C, as previously described [46], evaluating the decrease in absorbance at 340 nm linked to NADPH oxidation using a Biochrom Libra S60 spectrophotometer. The standard assay mixture contained 0.25 M sodium phosphate buffer pH 6.8, 0.18 mM NADPH, 2.4 M ammonium sulphate, 0.5 mM EDTA, and 4.7 mM d,l-glyceraldehyde. One unit of enzyme activity is the amount that catalyses the conversion of 1 μmol of substrate/min in the above assay conditions. For AR inhibition studies, L-idose (at the indicated concentrations) was used as substrate in the conditions described above.

PTP1B activity was determined as previously described [47]. Briefly, each assay was started by adding an aliquot of human recombinant PTP1B (1-302) in the assay buffer (0.075 M β,β-dimethylglutarate pH 7.0, containing 1 mM EDTA, 0.1 mM DTT, and *p*-nitrophenylphosphate (*p*-NPP)). All reactions were performed at 37 °C in a final volume of 1 mL. After the incubation time, the reactions were stopped by adding 2 mL of 0.1 M KOH to the samples. The concentration of the released *p*-nitrophenol was determined by measuring the absorbance at 400 nm in a 1 cm pathlength cuvette. One unit of enzyme activity is the amount that catalyses the conversion of 1 μmol of substrate/min in the above assay conditions.

### 4.5. Inhibition Studies

PE was dissolved in DMSO at a final concentration of 10 mM and added to the assay mixture containing 10 mU of purified AR (84 nM on the basis of a 34 KDa molecular weight) or 8 mU purified PTP1B (52 nM on the basis of a 36 KDa molecular weight). The reaction was started by the addition of the substrate. The DMSO concentration in the assays for AR determination was kept constant at 0.7 % (*v*/*v*) in order to avoid effects on AR activity [48]. The inhibitory effect of PE was determined by measuring the reaction rates in the presence of a fixed substrate concentration (2.5 mM *p*-NPP and 0.8 mM L-idose for PTP1B and AR, respectively) and increasing inhibitor concentrations (at least 14-16 different concentrations). The IC_50_ (the inhibitor concentration able to decrease the enzymatic activity up to 50%) values were determined by nonlinear regression analysis using Prism GraphPad 6.0 (GraphPad Software, San Diego, CA, USA), fitting experimental data to the following equation:

$$\frac{V_{i}}{V_{0}}\;=\;\frac{Max\;-\;Min}{1\;+\;{\left(\frac{x}{IC_{50}}\right)}^{slope}}\;+\;Min$$

where *Vi*/*V*_0_ represents the relative activity calculated in presence of each inhibitor concentration; “*Max*” and “*Min*” represent the maximum and minimum value of the activity, respectively; “*x*” is the concentration of the inhibitor; and “slope” represents the slope of the curve in the transition zone.

In order to define the mechanism of action of PE, reaction rates were measured in assay mixtures containing different L-idose concentrations (between 0.8 and 6 mM) in the case of AR, and different *p*-NPP concentrations (between 0.5 and 25 mM) in the case of PTP1B, in the absence and in the presence of different PE concentrations. Data were analysed by Lineweaver–Burk plots. The apparent dissociation constants *K_i_’* (for the ESI complex) and *K_i_* (for the EI complex) were determined from secondary plots of 1/^app^*V_max_* and ^app^K_M_/^app^*V_max_* as a function of the inhibitor concentration, respectively.

In order to evaluate the reversibility of the inhibitory action, 10 mU of purified AR was incubated for 90 min at 0 °C in the presence of PE (3 mM) in order to completely inhibit AR activity. The mixture was then extensively dialysed on Amicon ultrafiltration membrane (cut-off 3 KDa) against 10 mM sodium phosphate buffer, pH 7.0. After dialysis, the enzyme activity was again measured using 0.8 mM L-idose as substrate and compared to that of a mixture treated in the same conditions but in the absence of inhibitor. In order to evaluate the reversibility of the inhibitory action on PTP1B, 8 mU of PTP1B was incubated in the presence of a saturating concentration of PE (3 µM) and then incubated at 37 °C for 1 h. After this time, an aliquot of solution was withdrawn and extensively diluted in the assay buffer containing 2.5 mM of *p*-NPP. Residual activity of PTP1B was determined by calculating the amount of pNP released by measuring the absorbance of solution at 400 nm. 

### 4.6. Cell Cultures

HepG2 liver cell line was purchased from the American Type Culture Collection (ATCC, Manassas, VA, USA). HepG2 were routinely cultured in Dulbecco’s modified Eagle’s medium (DMEM)-high glucose (4500 mg/L) supplemented with 10% foetal bovine serum (FBS, Euroclone), 2 mM glutamine, 100 U/mL penicillin, and 100 μg/mL streptomycin. Cells were incubated at 37 °C in a humidified atmosphere with 5% CO_2_. 

### 4.7. Analysis of the Insulin Signalling Pathway

In order to evaluate the effect of PE on the insulin signalling pathway, HepG2 cells were seeded in 35-mm culture dishes and, after 24 hours, were incubated for 30 minutes with starvation medium (DMEM supplemented with 2 mM glutamine, 100 U/mL penicillin, and 100 μg/mL streptomycin) containing 25 μM of PE and/or 10 nM insulin (Humalog, lispro insulin). The medium was then removed, cells were washed with PBS, and lysed with sample buffer (2% SDS, 10% glycerol, 5% 2-mercaptoethanol, 0.002% bromophenol blue, and 0.0625 M Tris HCl, pH 6.8) for cellular protein extraction. Cellular extracts were boiled for 5 minutes, and then an aliquot (20 µg of protein) was analysed by SDS-PAGE and Western blot to evaluate both total and phosphorylated expression levels of Akt and IR. β-actin was used as a reference protein to normalise protein loading. 

### 4.8. Molecular Docking

Docking simulations were carried out with AutoDock (version 4.2.5.1, The Scripps Reasearch Institute, La Jolla, CA, USA) and AutoDock Tools (version 1.5.6rc3, The Scripps Reasearch Institute, La Jolla, CA, USA) [49] using the crystal structures PDB 1PTY and 3V36 structures for PTP1B and AR, respectively [50,51]. The grid map of the interaction energies for various atoms of the ligand with the macromolecule was calculated using AutoGrid software (version N 1.5.6., The Scripps Reasearch Institute, La Jolla, CA, USA). Specifically, for 1PTY, the grid search size was 126 × 100 × 90 points, with a spacing of 0.400 Å. The Lamarckian genetic algorithm (LGA) was used to identify potential binding pockets using the following parameters: The number of GA runs was 100, the number of individuals in the population was 150, the maximum number of energy evaluation was 250,000, and the maximum number of generations was 27,000. The LGA resulted in 100 ligand poses. These poses were clustered according to root-mean-square deviation (RMSD), with a similarity of 10 rms. Among all, five poses were selected from the obtained clusters. Graphical representations of the ligand, the protein, and the complexes were performed using UCSF Chimera [52]. 

The potential intermolecular interactions between the ligand and the protein were calculated automatically by LigPlot^+^ software (version 2.2, EMBL-EBI, Hinxton, Cambridgeshire, UK), which generated 2D-schematic representations of protein–ligand complexes displaying hydrogen bonds and hydrophobic interactions within the binding pocket [53,54].

### 4.9. Statistical Analysis of Data

Data obtained were analysed using OriginPro 2021 software (OriginLab Corporation, Northampton, MA, USA). Statistical significance between data was evaluated using ANOVA, followed by Tukey HSD. Data were checked for appropriate normality and homoscedasticity using the Shapiro–Wilk normality test and the Levene’s test, respectively. All tests were carried out in triplicate. 

## 5. Conclusions

In the past, many marine-derived molecules have been catalogued as PTP1B inhibitors [55], but very few of them have also been evaluated for their ability to inhibit AR, and none of those identified belong to the polyketide family [30]. To our knowledge, this is the first study showing that a polyketide extracted from a marine organism exhibits significant inhibitory activity towards PTP1B and AR. The evidence that PE possesses an intrinsic PTP1B/AR inhibitory activity suggests that it could be used as a natural lead to design multitarget drugs able to overcome insulin resistance and to counteract the onset of diabetic complications induced by the increased polyol pathway flux [56]. In conclusion, our data suggested that polyketides could be used as versatile scaffold structures for the synthesis of a new generation of potent and safe multitarget antidiabetic drugs. Considering the enormous biodiversity of the marine world, it cannot be excluded that further polyketide-like molecules will be identified in the future, enriching the database of marine molecules that act as inhibitors of PTP1B and AR. 

## Data Availability

Not applicable.

## Supplementary Materials

The following are available online at https://www.mdpi.com/article/10.3390/md19100535/s1, Figure S1: 1H-NMR spectrum of phosphoeleganin (PE) in CD3OD, Figure S2: Negative-ion HRESI mass spectrum of phosphoeleganin (PE), Figure S3: Determination of IC50 value for PE, and Figure S4: Dilution assay.
