# Supplementary material for: Dual Targeting of PTP1B and Aldose Reductase with Marine Drug Phosphoeleganin: A Promising Strategy for Treatment of Type 2 Diabetes
Source: Mar Drugs. 2021 Sep 24;19(10):535. doi: 10.3390/md19100535 (PMC8540617; doi:10.3390/md19100535)

Supplementary material

Dual targeting of PTP1B and Aldose Reductase with marine drug phosphoeleganin: a promising strategy for treatment of Type 2 Diabetes

Massimo Genovese[1,+], Concetta Imperatore[2,+], Marcello Casertano[2], Anna Aiello[2], Francesco Balestri[3], Lucia Piazza[3,‡], Marialuisa Menna[2,*], Antonella Del Corso[3,*] and Paolo Paoli[1,*]

[1] Department of Experimental and Clinical Biomedical Sciences, University of Florence, Viale Morgagni 50, 50134 Florence, Italy; m.genovese13@gmail.com

[2] Department of Pharmacy, University of Naples Federico II, Via D. Montesano 49, 80131 Napoli, Italy; cimperat@unina.it (C.I.); marcello.casertano@unina.it (M.C.); aiello@unina.it (A.A.)

[3] Department of Biology, Biochemistry Unit, University of Pisa, via S. Zeno, 51, 56123 Pisa, Italy; francesco.balestri@unipi.it (F.B.); l.piazza@studenti.unipi.it (L.P.)

* Correspondence: mlmenna@unina.it (M.M.); antonella.delcorso@unipi.it (A.D.C.); paolo.paoli@unifi.it (P.P.); Tel.: +39-081-678518 (M.M.); +39-050-2211454 (A.D.C.); +39-055-2751248 (P.P.)

† These authors contributed equally to this work.

‡ PhD student at the Tuscany Region Pegaso PhD School in Biochemistry and Molecular Biology, Italy

Table of contents

Figure S1. ^{1}H-NMR spectrum of phosphoeleganin (PE) in CD_3OD 2

Figure S2. Negative-ion HRESI mass spectrum of phosphoeleganin (PE) 2

Figure S3. Determination of IC_{50} value for PE 3

Figure S4. Dilution assay 3

Figure S1. ^{1}H NMR spectrum (700 MHz) of phosphoeleganin (PE) in CD_3OD

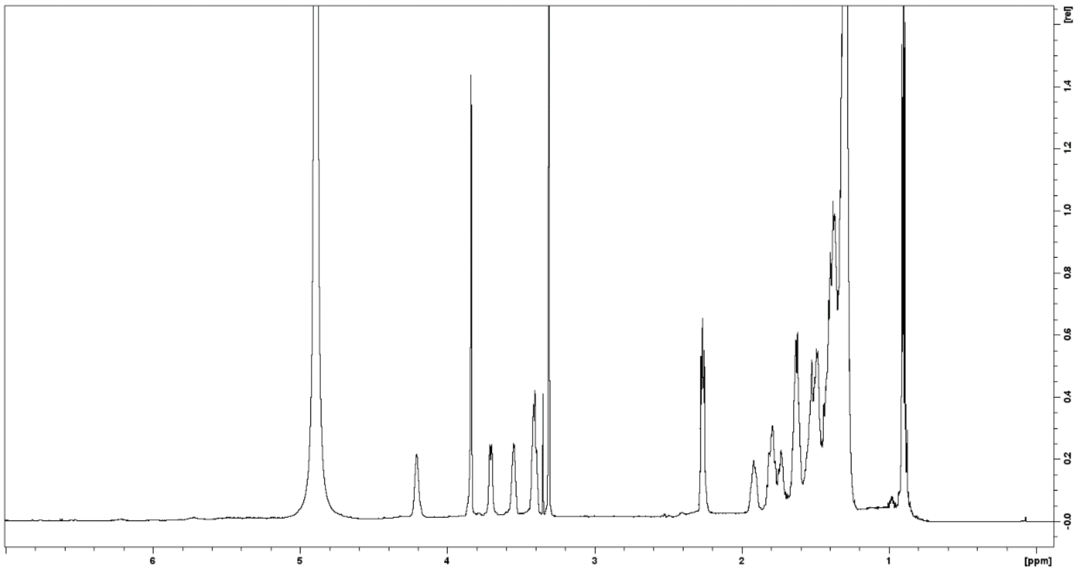

Figure S2. HRESI-MS spectrum of phosphoeleganin (PE)

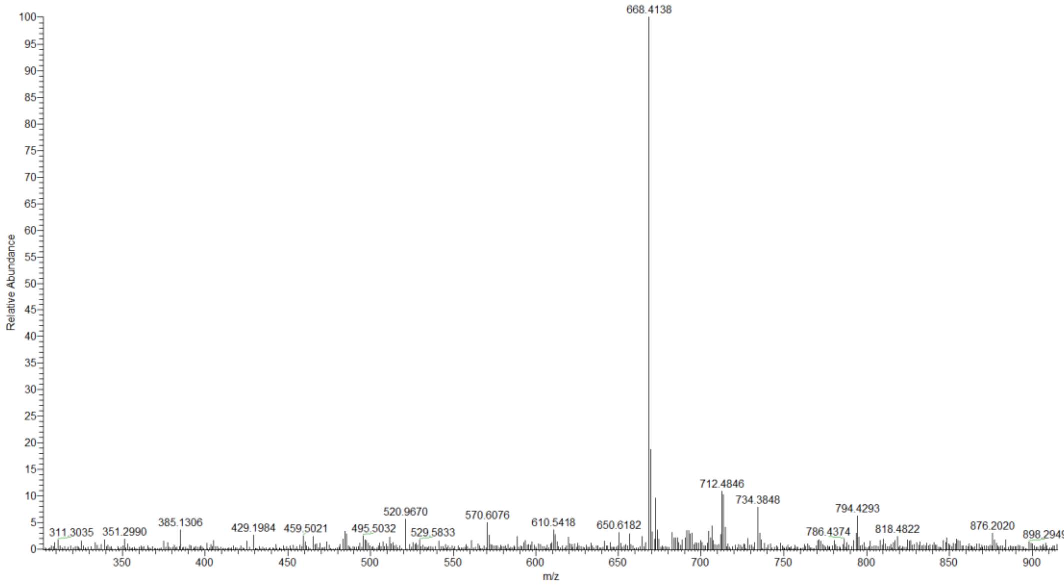

Figure S3. Determination of IC_{50} value for PE. An aliquot of PTP1B (8 mU) was incubated in the presence of increasing PE concentration in the assay buffer containing 2.5 mM pNPP. After 30 minutes, the reactions were stopped by adding 2 mL of 0.1 M KOH solution. The amount of p-NP released was determined using a spectrophotometer, measuring the absorbance of samples at 400 nm, using a 1cm path length cuvette. All tests were carried out in triplicate. Data obtained were normalized respect to control test and fitted using an opportune equation (see Material and method section).

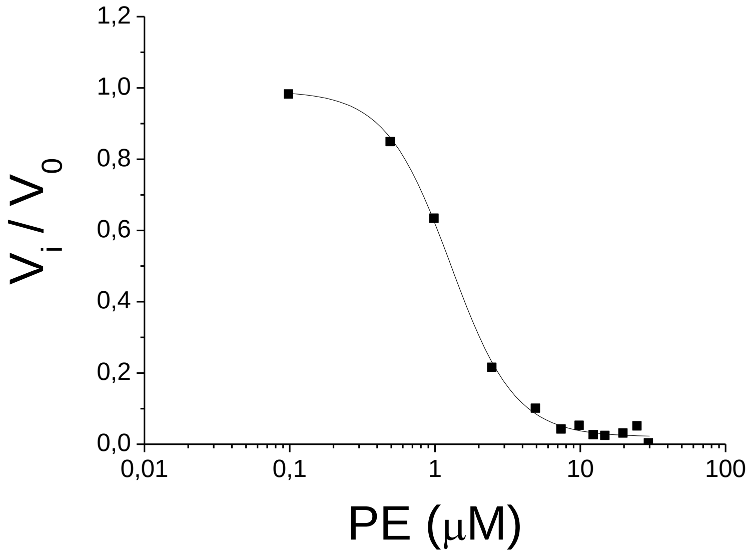

Figure S4. Dilution assay. The PTP1B was incubated in the presence of saturating concentration (3 μM) of PE, for 30 minutes at 37°C. Then, an aliquot of enzyme was diluted 400 folds in the assay buffer to evaluate the residual activity of enzyme. Tests were carried out in triplicate. Data obtained were normalized respect to control experiment carried out diluting PTP1B in the same solvent used to solubilize PE. Data show in the figure represent the mean value ± SEM (n=3).

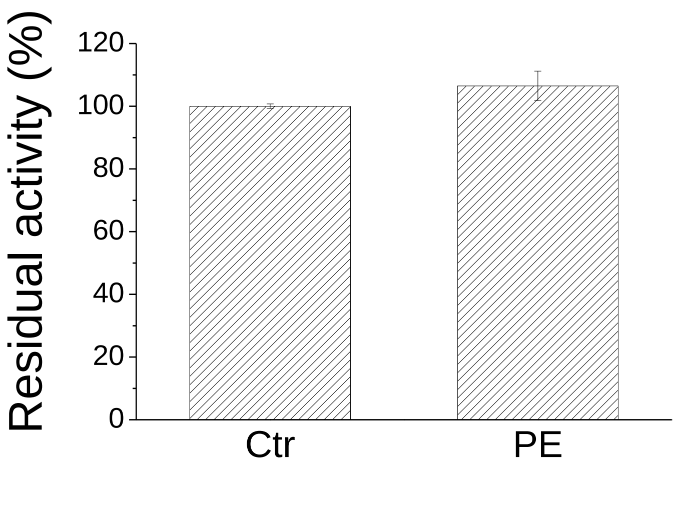

Supplement: Supplementary file 1 [file marinedrugs-19-00535-s001.zip › marinedrugs-1342413-supplementary.pdf]
